# Role of TRPV-1 in CGRP-mediated trigeminal sensitization

**DOI:** 10.1186/1129-2377-14-S1-P72

**Published:** 2013-02-21

**Authors:** D Chatchaisak, B Chetsawang, SM le Grand, P Govitrapong, A Srikiatkhachorn

**Affiliations:** 1 Research Center for Neuroscience, Institute of Molecular Biosciences, Mahidol Universit, Salaya, Nakhonpathom 73170, Thailand; 2 aResearch Center for Neuroscience, Institute of Molecular Biosciences, Mahidol Universit, Salaya, Nakhonpathom 73170, Thailand; 3 Department of Pathology, Faculty of Medicine, Chulalongkorn University, Thailand; 4 Deaprtment of Physiology, Faculty of Medicine, Chulalongkorn University, Bangkok, Thailand

## Introduction

Calcitonin gene-related peptide (CGRP) plays an important role in migraine pathogenesis. Transient receptor potential vanilloid-1 (TRPV1) is a cation channel which is expressed in sensory ganglia and can be activated by a number of pain inducing stimuli. The relationship between CGRP and TRPV-1 is still unclear.

## Objective

to investigate the functional role of CGRP on pain sensitization via the mediation of TRPV1 expression in rat trigeminal ganglion (TG).

## Materials and methods

CGRP (600 ng/kg) was intravenously injected to the Wistar rats (250-300 g). Rat trigeminal ganglia and brainstem were removed 45 and 60 minutes after CGRP or saline injection. The levels of CGRP, TRPV-1, phosphorylated PKC (p-PKC) and cyclic AMP responsive element-binding protein (CREB) in TG were determined by western blotting and by immunochemical and immunofluorescence staining. The c-fos level in trigeminal nucleus caudalis (TNC) was also determined.

## Results

Following CGRP injection, there was a significant increase in the amount of TRPV1, CGRP, p-PKC and CREB were observed in rat TG. In addition, an increase in c-fos levels was also determined in TNC of CGRP-treated rats indicating trigeminal nociception.

## Conclusion

These results indicate the role of TRPV-1 in CGRP-evoked trigeminal sensitization. This may explain the process of peripheral sensitization developed during the attack of migraine.
